# An Evolutionary History of Defensins: A Role for Copy Number Variation in Maximizing Host Innate and Adaptive Immune Responses

**DOI:** 10.3389/fimmu.2015.00115

**Published:** 2015-03-18

**Authors:** Lee R. Machado, Barbara Ottolini

**Affiliations:** ^1^Institute of Health and Wellbeing, School of Health, University of Northampton, Northampton, UK; ^2^Department of Cancer Studies, University of Leicester, Leicester, UK

**Keywords:** copy number variation, defensins, HIV, psoriasis, Crohn’s disease

## Abstract

Defensins represent an evolutionary ancient family of antimicrobial peptides that play diverse roles in human health and disease. Defensins are cationic cysteine-containing multifunctional peptides predominantly expressed by epithelial cells or neutrophils. Defensins play a key role in host innate immune responses to infection and, in addition to their classically described role as antimicrobial peptides, have also been implicated in immune modulation, fertility, development, and wound healing. Aberrant expression of defensins is important in a number of inflammatory diseases as well as modulating host immune responses to bacteria, unicellular pathogens, and viruses. In parallel with their role in immunity, in other species, defensins have evolved alternative functions, including the control of coat color in dogs. Defensin genes reside in complex genomic regions that are prone to structural variations and some defensin family members exhibit copy number variation (CNV). Structural variations have mediated, and continue to influence, the diversification and expression of defensin family members. This review highlights the work currently being done to better understand the genomic architecture of the β-defensin locus. It evaluates current evidence linking defensin CNV to autoimmune disease (i.e., Crohn’s disease and psoriasis) as well as the contribution CNV has in influencing immune responses to HIV infection.

## Introduction

The defensins represent a class of cationic antimicrobial peptides that play pivotal roles in innate and adaptive immunity as well as roles in non-immunological processes. They constitute an ancient and diverse gene family, present in most multicellular organisms ranging, from plants, fungi, insects, mollusks, and arachnids to mammals, including humans. During their evolutionary history, defensins have become highly diversified and have acquired novel functions in different species. Defensins have evolved to be highly efficient in their antimicrobial responses to a vast array of pathogens.

The term “Defensins” was coined in 1985 after granule rich sediments were purified from human and rabbit neutrophils. This resulted in the characterization of the primary structure of the first six neutrophils defensins (later known as α-defensins) (1–3). These early studies highlighted the structural hallmarks of defensins: that is, despite poor sequence identity across family members, all defensins possesses a highly conserved motif of six cysteine residues that is key to their antimicrobial function. Subsequently, peptides with similar structure were discovered in the early 1990s in bovine (4) and mouse airway first (5) and subsequently in the human intestinal epithelium (6), and became known as β-defensins. The recent ability to interrogate genomic and proteomic data from a diverse array of species allowed the discovery and characterization of further members of the defensin gene family, intensifying interest in unveiling the roles of defensins in physiological and pathological processes.

This review will primarily focus on the role of β-defensins in innate and adaptive immunity. We will highlight the methods currently employed to study the genomic architecture of this multifunctional gene family and how complex genetic variation has an impact on defensin host inflammatory responses.

## Structure of β-Defensins

The β-defensin family members have poor sequence similarity, suggesting their antimicrobial activity is independent of their primary structure. Nuclear magnetic resonance (NMR) data have been used to evaluate the 3D structure of hBD1, hBD2, and hBD3 (7, 8). These data confirm a high degree of similarity in their tertiary structures, despite their diverged amino acid sequences. The major element of the mature peptides secondary structure is represented by three β-strands arranged in an antiparallel sheet. The strands are held together by the three intramolecular disulfide bonds, formed between the six cysteines. The order of the disulfide bridges can vary, characterizing each family member. The amino-terminal region contains a short α-helical loop (which is absent in α-defensins). α-helical structures are common for protein regions that are incorporated into cell membranes and it has been proposed that this region of the β-defensin protein may anchor to bacteria cell walls (9). This is supported by the presence of two sites under positive selection located in the N-terminal region that may contribute to β-defensin functional diversity (10).

Defensins do not appear to present a distinct hydrophobic core or a common pattern of charged or hydrophobic residues on the protein surface. This suggests peptide folding is driven and stabilized by disulfide bond formation alone. Moreover, the characteristic β-defensin 3D structure can be preserved and accommodates residues with different properties at most other positions. The first five amino acids of the mature peptide sequence are vital for correct protein folding under oxidative conditions. This favors the formation of the correct disulfide bonded pattern through the creation of a key intermediate (11).

## The Evolution and Divergent Roles of β-Defensins

The evolutionary relationship between vertebrate and non-vertebrate defensins is still unclear; however, phylogeny indicates that a primordial β-defensin is the common ancestor of all vertebrate defensins and this gene family expanded throughout vertebrate evolution (12). This hypothesis is supported by the discovery of β-defensin-like genes in phylogenetically distant vertebrates, including reptiles (13), birds (14), and teleost fishes (15). α-defensins are mammalian specific genes, and in humans α-defensin genes and different β-defensin genes are present on adjacent loci on chromosome 8p22–p23. The organization of this cluster is consistent with a model of multiple rounds of duplication and divergence under positive selection from a common ancestral gene that produced a cluster of diversified paralogous (16, 17). This expansion occurred before the divergence of baboons and humans ~23–63 million years ago (18, 19). The present-day β-defensins probably evolved before mammals diverged from birds generating α-defensins in rodents, lagomorphs, and primates after their divergence from other mammals (20). Recent evidence suggests convergent evolution of β-defensin copy number (CN) in primates, where independent origins have been sponsored by non-allelic homologous recombination between repeat units. For rhesus macaques this resulted in only a 20 kb copy number variation (CNV) region containing the human ortholog of human β-defensin 2 gene. In humans, recent work suggests a repeat unit of 322 kb containing a number of β-defensin genes (21).

Defensin family members possess a plethora of non-immune activities and it is instructive to provide some examples of the diverged nature of defensins function. Some members of the β-defensin family have an important role in mammalian reproduction [reviewed in Ref. (22)]. For example, there are five human defensin genes (*DEFB125*–*DEFB129*) clustered on chromosome 20, which are highly expressed in the epithelial cell layer of the epididymal duct, which secretes factors responsible for sperm maturation (23). Moreover, human *DEFB118* was shown to be a potent antimicrobial peptide able to bind to sperm, probably providing protection from microorganisms present in the sperm ducts (24). It is noticeable how in long tailed macaque (*Macaca fascicularis*) and in rhesus macaque (*Macaca mulatta*), there is a similar β-defensin, called *DEFB126*, which is the principal protein that coats sperm (25); this coating is lost in the oviduct allowing fertilization to occur. In support of this, the deletion of a cluster of nine beta defensin genes in a mouse model, resulted in male sterility (26). In human studies, a common mutation in *DEFB126* has been shown to impair sperm function and fertility (27).

In a second example, recent studies have suggested that some β-defensin gene products including hBD1 and hBD3, can interact with a family of melanocortin receptors, modulating pigment expression in dogs and possibly in humans (28). Typically, there are two genes that control the switching of pigment types: the melanocortin receptor 1 (*Mc1r*) and *Agouti*, encoding a ligand for the Mc1r, which inhibits Mc1r signaling. Mc1r activation determines production of the dark pigment eumelanin exclusively, whereas Mc1r inhibition causes production of the lighter pigment pheomelanin. In dogs, it was discovered that a mutation in the canine *DEFB103* is responsible for the dominant inheritance of black coat color, which does not signal directly through Mc1r; this insight revealed a previously uncharacterized role of β-defensins in controlling skin pigmentation. Further studies have been conducted on human melanocytes, discovering a novel role of hBD3 as an antagonist of the α-melanocyte-stimulating hormone (α-MSH, a known agonist of Mc1r, which stimulates cAMP signaling to induce eumelanin production). As hBD3 is produced by keratinocytes, it can act as a paracrine factor on melanocytes modulating α-MSH effects on human pigmentation and consequently responses to UV (29). Moreover, it is known that melanocortin receptors are also involved in inflammatory and immune response modulation (30).

## Expression of β-Defensins

Different β-defensins are present in different epithelial and mucosal tissues and can be constitutively expressed or induced in response to various stimuli (31–52) (Table S1 in Supplementary Material). Their anatomical distribution clearly reflects their ability to neutralize different pathogens and they are more abundant at sites prone to the microbial infections they are specific for. For example, hBD2 is strongly expressed in lung (53); hBD4 is highly expressed in the stomach and testes (54), and hBD3 in the skin and tonsillar tissue (55). hBD1–hBD4 are expressed in the respiratory tract, with constitutive expression of hBD1 (56) and inducible expression of hBD2–hBD4 in response to inflammation or infection (57). In keratinocytes, there is constitutive mRNA expression of hBD1; conversely hBD2 expression is induced by lipopolysaccharides (LPS) or other bacterial epitopes in combination with interleukin-1β, released by resident monocyte-derived cells. hBD3 and hBD4 are inducible by stimulation with tumor necrosis factor (TNF), toll-like receptor ligands, interferon (IFN)-γ, or phorbolmyristate acetates (58). hBD3 is also induced in response to local release of surface-bound epidermal growth factor receptor (EGFR) ligands via activation of metalloproteinases (59, 60).

## Antimicrobial Activity of β-Defensins

The most studied function for β-defensins is their direct antimicrobial activity, through permeabilization of the pathogen membrane. Their exact mechanism of action is incompletely understood and two different models have been proposed. The first is a carpet model, where several antimicrobial peptides opsonize the pathogen surface bringing about necrosis, possibly disrupting the electrostatic charge across the membrane (61). The latter is a pore model, with several peptides oligomerizing and forming pore-like membrane defects that allow efflux of essential ions and nutrients (55).

Defensins *in vitro* are active against gram negative and positive bacteria, unicellular parasites, viruses, and yeast. Cationic peptides including β-defensins are attracted to the overall net negative charge generated by the outer envelope of Gram negative bacteria by phospholipids and phosphate groups on LPS and to the teichoic acid present on the surface of Gram positive bacteria.

β-defensins also possess anti-viral activity, interacting directly with the virus and indirectly with its target cells. Noticeably, in mammals, β-defensins are also produced by the oral mucosa and they are active against HIV-1 virus: in particular, hBD1 is constitutively expressed whereas the presence of a low HIV-1 viral load can stimulate the expression of hBD2 and hBD3 gene products through direct interaction with the virus. More specifically, hBD2 has been shown to down-regulate the HIV transcription of early reverse-transcribed DNA products (62) and hBD2 and hBD3 can mediate CXCR4 down-regulation (but not CCR5) and internalization in immuno-stimulated peripheral blood mononuclear cells (63). This mechanism diminishes the chances of infection (64) and with other salivary gland components, could help to explain the oral mucosal natural resistance to HIV infection. hBD3 also possesses an inhibitory effect on the influenza virus blocking the fusion of the viral membrane with the endosome of the host cell, through cross linking of the viral glycoproteins (65).

Defensins have evolved to maximize their protective role, showing an extraordinary adaptation to different environmental challenges: for instance, plant defensins are particularly active against fungal infections [reviewed in Ref. (66)], slowing down hyphal elongation, and some of them also evolved to gain an α-amylase inhibitory activity that can confer protection against herbivores (67, 68).

## Immune Modulatory Activity of β-Defensins

A role for defensins in pro-inflammatory responses and more recently immunosuppression [reviewed in Ref. (69)] has been delineated over the last two decades. An initial important observation was that β-defensins can recruit immature dendritic cells and memory T cells to sites of infection and/or inflammation providing a link between the innate and adaptive arms of the immune system. A mechanism for this was provided by Oppenheim’s group where they demonstrated that natural and recombinant hBD2 could chemoattract human immature dendritic cells and memory T cells *in vitro* in a dose-dependent manner. This response was inhibited with the Gαi inhibitor pertussis toxin and suggested the possible involvement of a chemokine receptor(s), which was confirmed using anti-CCR6 blocking antibodies.

T_H_17 cells express CCR6 and respond to β-defensins chemoattractant action. Furthermore, T_H_17 cytokines (i.e., IL-17 and IL-22) induce expression of defensins from relevant cell types including primary keratinocytes potentially resulting in an amplification of T_H_17 responses (70). Increased T_H_17 levels have been reported in different autoimmune diseases, such as multiple sclerosis (71), rheumatoid arthritis (72), and psoriasis (73), implicating β-defensin expression in autoimmunity. Given the role of defensins in chemoattracting monocytes and macrophages and the lack of CCR6 on these cell types other receptors were investigated that might mediate this chemoattractant activity. This resulted in the identification of CCR2 as a receptor for hBD2, hBD3, and their mouse orthologs (mBD4 and mBD14) (74).

In addition to signaling through chemokine receptors, defensins have been shown to function through toll-like receptors (75, 76). hBD2 has been shown to be a natural ligand for the toll-like-receptor-4 (TLR-4), present on immature DCs, up-regulating co-stimulatory molecules and leading to DC maturation, and on CD4^+^ T cells, possibly stimulating their proliferation and survival (77). On bone marrow-derived macrophages pre-treated with a recently identified mBD14 (78), TLR restimulation of these cells resulted in enhanced expression of pro-inflammatory mediators that was Gi protein dependent but independent of CCR2 or CCR6 signaling pathways (79).

## β-Defensin Copy Number Variation and Disease Association Studies

**Figure 1:**
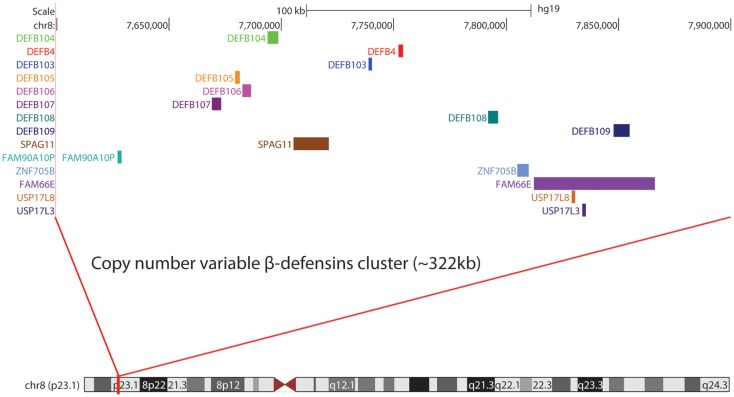
**Genome assembly of β-defensin repeat unit at 8p23.1**.

In humans, β-defensins genes are organized into three main clusters at 8p23.1, 20p13, and 20q11.1, with another likely small cluster on chromosome 6p12 (80). At 8p23.1, a number of β-defensins are found on a repeat unit that is typically present at 2–8 copies in the population, with a modal CN of 4. Each chromosome 8 copy can contain 1–8 copies of the repeat unit. The mutation rate at this locus is extremely fast (~0.7% per gamete) (81), indicative of the high level of plasticity in this genomic region. One-copy individuals are extremely rare (82, 83), and suggest that the presence of a null allele might be deleterious and selected against. At the other end of the *DEFB*, CN spectrum lies a proportion of high-copies individuals (9–12 copies) with a cytogenetically visible CN amplification at 8p23.1 that has no phenotypic effect (84). These first experimental observations ignited further interest into the chromosome 8 *DEFB* cluster. Within the repeat unit there is *DEFB4*, *DEFB103*, *DEFB104*, *DEFB105*, *DEFB106*, *DEFB107*, *SPAG11*, and *PRR23D1* (21, 85) (Figure 1). The variation in the number of repeat units between individuals in the population and likely sequence variation between copies suggests that CNV of defensins may play a role in modulating defensin expression (86, 87) and function. The consequences of CNV have been explored for a number of years and may include increased gene product, the production of fusion genes, the formation of extra coding domains, or a position effect that alters expression of the gene product (88). This extensive structural genome variation in humans is particularly pertinent to diseases where defensins may be implicated in their pathology. This includes a number of autoimmune and infectious diseases (Table 1).

**Table 1:** **Summary of β-defensin CNV studies**.

*DEFB* cluster CN calls per diploid genome	Sample size	Methods used for CN calling	Association study?	Findings	Reference
2–12	90 Controls	MAPH	No	Average CN distribution of 2–7 for controls	(89)
	12 Related individuals from 3 families with chr8p23 euchromatic variant (EV)	SQ-FISH		Average CN distribution of 2–7 for EV carriers	
2–8	27 Unrelated samples	qPCR	No	Concordant CN for *DEFB4* and *DEFB103*	(90)
2–10	355 Patients with cystic fibrosis	MAPH	Cystic fibrosis	*DEFB* CN is not associated with cystic fibrosis	(91)
	167 UK controls	
2–7 for *DEFB4*	44 Samples	qPCR	No	Discordant CN for *DEFB4*, *DEFB103* and *DEFB104*	(92)
2–10	250 CD patients	Array-CGH	Crohn’s disease	<3 copies associated with CD (OR = 3.06)	(93)
	252 Controls	qPCR	
2–12	498 Cases	MAPH	Psoriasis	Higher CN associated with psoriasis	(94)
	305 Controls	PRT		RR = 1.69 >6 copies	
2–8	>800 Samples	MAPH/REDVR, MLPA and array-CGH. All validated through PRT	No	PRT is a reliable method for CNV analysis	(95)
2–9	42 Samples	MLPA	No	Strict copy number concordance for all genes in the chr8p23.1 *DEFB* cluster	(96)
1–12	208 Offspring from 26 CEPH families	PRT Microsatellite analysis	No	Fast germline copy number recombination of DEFB cluster (~0.7% per gamete)	(81)
1–12 in CD patients	466 CD patients 329 Controls	qPCR	Crohn’s disease	>4 copies associated with CD (OR = 1.54)	(97)
2–9 in controls		
1–10	1000 Crohn’s disease (CD) patients	PRT on all samples	Crohn’s disease	*DEFB* copy number is not associated with CD (higher accuracy in CN calling and a larger cohort compared with previous studies on CD)	(82)
	500 Controls	qPCR on 625 samples	
1–9	1056 Individuals from the HGDP–CEPH panel	PRT	No	Recent selection of high-expressing *DEFB103* gene copy in East Asia	(83)
1–9	1002 Ethiopian and Tanzanian HIV and HIV/TB patients	PRT	HIV viral load in HIV-only and HIV/TB patients	Increased HIV load prior to HAART (*P* = 0.005) and poor immune reconstitution following initiation of HAART (*P* = 0.003)	(98)
2–7	543 SLE patients	PRT	Systemic lupus erythematosus	Higher CN associated with SLE and AASV (SLE OR = 1.2; AASV OR = 1.5)	(99)
	112 AASV patients	515 samples validated with REDVR	ANCA associated small vasculitis (AASV)	
	523 Controls	
2–8	70 PDAC patients	MLPA	Pancreatic ductal adenocarcinoma (PDAC)	Protective effect of high *DEFB* CN against PDAC (Fisher’s exact test *P* = 0.027)	(100)
	60 CP patients		Chronic pancreatitis (CP)	
	392 Controls	
1–9	2343 Samples (689 children and 1149 adults)	PRT	Asthma	*DEFB* CN is not associated with lung function in the general population (OR = 0.89)	(101)
			Chronic obstructive pulmonary disease (COPD)	
2–9	113 Otitis media prone children 259 Controls	PRT	Susceptibility to otitis media	*DEFB* CN associated with nasopharyngeal microbiota composition (with respect to the three predominant pathogens for otitis media: *S. pneumoniae*, *M. catarrhalis*, and *H. influenzae*	(102)

*AASV, ANCA associated small vasculitis; array-CGH, array comparative genomic hybridization; CD, Crohn’s disease; CEPH, Centre d’Etude du polymorphisme humain DNA panel; COPD, chronic obstructive pulmonary disease. CP, chronic pancreatitis; HAART, highly active anti-retroviral therapy; HGDP, human genome diversity cell line panel; MAPH, multiplex amplifiable probe hybridization; MLPA, multiplex ligation-dependent probe amplification; PDAC, pancreatic ductal adenocarcinoma; PRT, paralog ratio test; REDVR, restriction enzyme digest variant ratio; SLE, systemic lupus erythematosus; SQ-FISH, semi-quantitative fluorescence *in situ* hybridization; TB, tuberculosis*.

Mapping of the β-defensin CNV region has been challenging but recent data fixes the minimal length of the CNV at 157 kb (103) and a recent study using high density array comparative genomic hybridization combined with paralog ratio test (PRT) assays suggests it may be as large as 322 kb (21). Because of the extensive CNV of defensins, robust methods are required to accurately interrogate CN states in disease cohorts. Various locus specific techniques for CN determination have been utilized including multiplex amplifiable probe hybridization (MAPH) (104), multiple ligation probe amplification (MLPA) (105), and PRT (95). The advantage of such techniques is the ability to obtain data that clusters around integer CNs providing a high degree of concordance between the methods and confidence in the CN obtained. Association studies investigating some CNVs (i.e., *CCL3L1/CCL4L2* in HIV) have provided conflicting results as the methods used did not generate data that clustered around integer CN values (106, 107). In some cases, initial findings have been replicated in subsequent studies that have utilized more robust methods (108).

In early association studies of multi-allelic CNV and disease, CNV of defensins was implicated in psoriasis. Individuals with more than five β-defensin copies presented a fivefold increased risk of developing psoriasis when compared to two copy individuals. In addition, there was a direct correlation between the number of copies and relative risk (odds ratio of 1.32) (94). This association was replicated (although with reduced odds ratio) in a subsequent study (109). In the case of an autoimmune condition, such as psoriasis, high CN may contribute to the strong induction of hBD2 and hBD3, conferring protection from bacterial infections of the psoriatic lesions (110).

Another disease strongly linked with defensin expression is Crohn’s disease (CD) where it has been demonstrated that reduced Paneth cell expression of defensins in the ileum results in ileal CD. Therefore, defensin expression at this site may be important in maintaining the mucosal microbiota. *NOD2* has been strongly implicated in the pathogenesis of CD from GWAS (111) giving a 17.1-fold increased risk for CD in homozygous or compound heterozygous individuals. *NOD2* is a nod like family receptor (NLR) member that controls expression of defensins in CD. Polymorphisms in *NOD2* result in reduced α-defensin expression and exacerbated disease. Polymorphism of the *DEFB1* (non-CNV gene) promoter has been associated with CD (112). So is there a role for CNV in CD? Previous studies indicated that α-defensin CN may be important (113). However, recent work that accurately measured CN using PRTs to determine CN of *DEFA1A3* determined that a SNP (rs4300027) is associated with *DEFA1A3* CN in Europeans (114). This SNP was then used to indirectly interrogate GWAS data and suggested that α-defensins CNV may not be important in CD. A similar outcome was obtained with β-defensin CN whereupon accurate measurement, there was no association with the CD (82) in contrast to previous reports (93, 97). These results, however, do not exclude the role of α and β-defensin expression in the pathogenesis of CD but suggest that the individuals CN state may not be important in this context.

Given the suspected anti-viral role of defensins, it was suggested that defensin CNV may be important in host responses to HIV infection. There are a number of conflicting reports of the association between defensin CN and HIV infection (114–116). A surprising finding from a cohort study that evaluated two sub-Saharan populations with HIV-1 or HIV-1/tuberculosis coinfection was that high CN of β-defensins did not result in the predicted low viral load and did not improve immune reconstitution in patients (98). The converse was found suggesting that the immune modulatory properties of defensins may be subverted during HIV-1 infection. A model suggested to explain this apparently paradoxical result was that high CN may promote increased recruitment of CCR6 expressing cell types that are highly permissive for HIV-1 infection thus amplifying the foci of HIV-1 infection.

## Conclusion

Defensins play a key role in pathogen host interactions and are at the interface of innate and adaptive immunity. The complex genetic variation that underlies the evolutionary history of defensins and their biology is gradually being elucidated, suggesting defensin CNV is an important contributor to maximizing the host innate and adaptive response. The history of the defensin gene family is particularly paradigmatic given that many CNV loci in the human genome host immunity genes. Further studies should be conducted to better understand the genomic architecture of multi-allelic CNVs. This will aid the development of robust assays that evaluate the overall impact that CNV has on and both physiological and pathological mechanisms of immunity.

## Conflict of Interest Statement

The authors declare that the research was conducted in the absence of any commercial or financial relationships that could be construed as a potential conflict of interest.

## Supplementary Material

The Supplementary Material for this article can be found online at http://www.frontiersin.org/Journal/10.3389/fimmu.2015.00115/abstract
