# Supplementary material for: An Evolutionary History of Defensins: A Role for Copy Number Variation in Maximizing Host Innate and Adaptive Immune Responses
Source: Front Immunol. 2015 Mar 18;6:115. doi: 10.3389/fimmu.2015.00115 (PMC4364288; doi:10.3389/fimmu.2015.00115)
Supplement: Supplementary file 1 [file Table_1.PDF]

**Gene**	**Peptide**	**Tissue distribution**	**Synthesis and regulation**
DEFB4	Human β-defensin 2 (HBD2)	Oral (3) and nasal mucosa (4), lungs (2), plasma (5), salivary glands (3), small and large bowel (6), stomach (7), eyes (8), skin (9), and kidney with chronic infections (10).	Inducible in response to viruses (11), bacteria (6), lipopolysaccharide (3,12), peptidoglycan (13), lipoproteins (14), cytokines (IL1α (6), IL-1β (15), TNF (16)), PMA (17), IFN-γ (HBD3 only, and growth factors.<br>TLR2-mediated expression of HBD2 (18).<br>Constitutive expression on ocular surface (HBD3) (8). HBD3 CSE inducible (19).
DEFB103	Human β-defensin 3 (HBD3)	Leukocytes, placenta, testis, heart, skeletal muscle (20), urinary tract (21)	
DEFB104	Human β-defensin 4 (HBD4)	Gastric antrum, oral mucosa (22) and testis	Constitutive or inducible in response to PMA (17), TNF-α (17) and bacteria.<br>Constitutive mRNA expression in gingival keratinocytes (22).
DEFB105	Human β-defensin 5 (HBD5)	Testis	*In vitro* antimicrobial activity against *E.coli* but not *S.aureus* (23).<br>Constitutive mRNA expression in testis (24).<br>HBD5 CSE inducible (19).
DEFB106	Human β-defensin 6 (HBD6)	Testis , lung (25)	
DEFB107	Human β-defensin 7 (HBD7)	Oral mucosa (22), testis	Constitutive mRNA expression in gingival keratinocytes (22).<br>Constitutive mRNA expression in testis (24).
DEFB108	Human β-defensin 8 (HBD8)	Lung, oral mucosa (22)	Inducible by IL-1β (1) and *Candida spp* (22).<br>Constitutive mRNA expression in testis (24).
DEFB109	Human β-defensin 9 (HBD9)	Oral mucosa (22), lung, ocular surface (8)	Constitutive mRNA expression in gingival keratinocytes (22).<br>Constitutive expression on ocular surface (8).<br>mRNA almost ubiquitously expressed (25).<br>CSE inducible (19).

Table S1. Summary of β-defensin tissue distribution, synthesis and regulation

References

1. Schibli DJ, Hunter HN, Aseyev V, Starner TD, Wiencek JM, McCray PB, Tack BF, Vogel HJ. The solution structures of the human beta-defensins lead to a better understanding of the potent bactericidal activity of HBD3 against Staphylococcus aureus. *The Journal of biological chemistry* (2002) **277**:8279–89. doi:10.1074/jbc.M108830200

2. Bals R, Wang X, Wu Z, Freeman T, Bafna V, Zasloff M, Wilson JM. Human beta-defensin 2 is a salt-sensitive peptide antibiotic expressed in human lung. *The Journal of clinical investigation* (1998) **102**:874–80. doi:10.1172/JCI2410

3. Mathews M, Jia HP, Guthmiller JM, Losh G, Graham S, Johnson GK, Tack BF, McCray PB. Production of β-defensin antimicrobial peptides by the oral mucosa and salivary glands. *Infection and immunity* (1999) **67**:2740–2745.

4. Chen P-H, Fang S-Y. Expression of human β-defensin 2 in human nasal mucosa. *European Archives of Oto-Rhino-Laryngology and Head & Neck* (2004) **261**:238–241.

5. Hiratsuka T, Nakazato M, Date Y, Ashitani J, Minematsu T, Chino N, Matsukura S. Identification of human β-defensin-2 in respiratory tract and plasma and its increase in bacterial pneumonia. *Biochemical and biophysical research communications* (1998) **249**:943–947.

6. O'Neil DA, Porter EM, Elewaut D, Anderson GM, Eckmann L, Ganz T, Kagnoff MF. Expression and regulation of the human β-defensins hBD-1 and hBD-2 in intestinal epithelium. *The Journal of Immunology* (1999) **163**:6718–6724.

7. Hamanaka Y, Nakashima M, Wada A, Ito M, Kurazono H, Hojo H, Nakahara Y, Kohno S, Hirayama T, Sekine I. Expression of human β-defensin 2 (hBD-2) inHelicobacter pylori induced gastritis: antibacterial effect of hBD-2 against Helicobacter pylori. *Gut* (2001) **49**:481–487.

8. Otri AM, Mohammed I, Al-Aqaba MA, Fares U, Peng C, Hopkinson A, Dua HS. Variable expression of human Beta defensins 3 and 9 at the human ocular surface in infectious keratitis. *Investigative ophthalmology & visual science* (2012) **53**:757–61. doi:10.1167/iovs.11-8467

9. Harder J, Bartels J, Christophers E, Schroder JM. A peptide antibiotic from human skin. *nature* (1997) **387**:861.

10. Lehmann J, Retz M, Harder J, Krams M, Kellner U, Hartmann J, Hohgräwe K, Raffenberg U, Gerber M, Loch T. Expression of human beta-defensins 1 and 2 in kidneys with chronic bacterial infection. *BMC infectious Diseases* (2002) **2**:20.

11. Sun L, Finnegan CM, Kish-catalone T, Blumenthal R, Garzino-demo P, La GM, Maggiore T, Berrone S, Kleinman C, Wu Z, et al. Human ␤ -Defensins Suppress Human

Immunodeficiency Virus Infection : Potential Role in Mucosal Protection †. (2005) **79**:14318–14329. doi:10.1128/JVI.79.22.14318

12. Diamond G, Russell JP, Bevins CL. Inducible expression of an antibiotic peptide gene in lipopolysaccharide-challenged tracheal epithelial cells. *Proceedings of the National Academy of Sciences* (1996) **93**:5156–5160.

13. Kumar A, Zhang J, Fu-Shin XY. Innate immune response of corneal epithelial cells to Staphylococcus aureus infection: role of peptidoglycan in stimulating proinflammatory cytokine secretion. *Investigative Ophthalmology & Visual Science* (2004) **45**:3513–3522.

14. Birchler T, Seibl R, Büchner K, Loeliger S, Seger R, Hossle JP, Aguzzi A, Lauener RP. Human Toll-like receptor 2 mediates induction of the antimicrobial peptide human beta-defensin 2 in response to bacterial lipoprotein. *European journal of immunology* (2001) **31**:3131–3137.

15. McDermott AM, Redfern RL, Zhang B, Pei Y, Huang L, Proske RJ. Defensin expression by the cornea: multiple signalling pathways mediate IL-1β stimulation of hBD-2 expression by human corneal epithelial cells. *Investigative Ophthalmology & Visual Science* (2003) **44**:1859–1865.

16. Harder J, Meyer-Hoffert U, Teran LM, Schwichtenberg L, Bartels J, Maune S, Schroder J-M. Mucoid Pseudomonas aeruginosa, TNF-α, and IL-1 β, but Not IL-6, Induce Human β-Defensin-2 in Respiratory Epithelia. *American journal of respiratory cell and molecular biology* (2000) **22**:714–721.

17. Vankeerberghen A, Nuytten H, Dierickx K, Quirynen M, Cassiman J-J, Cuppens H. Differential induction of human beta-defensin expression by periodontal commensals and pathogens in periodontal pocket epithelial cells. *Journal of periodontology* (2005) **76**:1293–1303.

18. Kumar A, Zhang J, Yu F-SX. Toll-like receptor 2-mediated expression of β-defensin-2 in human corneal epithelial cells. *Microbes and Infection* (2006) **8**:380–389.

19. Semlali A, Witoled C, Alanazi M, Rouabhia M. Whole cigarette smoke increased the expression of TLRs, HBDs, and proinflammory cytokines by human gingival epithelial cells through different signaling pathways. *PloS one* (2012) **7**:e52614.

20. García J-R, Jaumann F, Schulz S, Krause A, Rodríguez-Jiménez J, Forssmann U, Adermann K, Klüver E, Vogelmeier C, Becker D. Identification of a novel, multifunctional β-defensin (human β-defensin 3) with specific antimicrobial activity. *Cell and tissue research* (2001) **306**:257–264.

21. Lüthje P, Hirschberg AL, Brauner A. Estrogenic action on innate defense mechanisms in the urinary tract. *Maturitas* (2014) **77**:32–36.

22. Premratanachai P, Joly S, Johnson GK, McCray PB, Jia HP, Guthmiller JM. Expression and

regulation of novel human β-defensins in gingival keratinocytes. *Oral microbiology and immunology* (2004) **19**:111–117.

23. Huang L, Ching CB, Jiang R, Leong SSJ. Production of bioactive human beta-defensin 5 and 6 in< i> Escherichia coli</i> by soluble fusion expression. *Protein expression and purification* (2008) **61**:168–174.

24. Semple CAM, Rolfe M, Dorin JR. Duplication and selection in the evolution of primate b-defensin genes. *Genome Biol* (2003) **4**:R31.

25. Kao CY, Chen Y, Zhao YH, Wu R. ORFeome-based search of airway epithelial cell-specific novel human [beta]-defensin genes. *American journal of respiratory cell and molecular biology* (2003) **29**:71–80. doi:10.1165/rcmb.2002-0205OC
